# Genetic Validation of* Leishmania donovani* Lysyl-tRNA Synthetase Shows that It Is Indispensable for Parasite Growth and Infectivity

**DOI:** 10.1128/mSphereDirect.00340-17

**Published:** 2017-08-30

**Authors:** Sanya Chadha, N. Arjunreddy Mallampudi, Debendra K. Mohapatra, Rentala Madhubala

**Affiliations:** a School of Life Sciences, Jawaharlal Nehru University, New Delhi, India; b Natural Products Chemistry Division, CSIR-Indian Institute of Chemical Technology, Hyderabad, India; University at Buffalo; McGill University; University of Washington

**Keywords:** *Leishmania donovani*, lysyl-tRNA synthetase, drug targets, genetic validation

## Abstract

*Leishmania donovani* is a protozoan parasite that causes visceral leishmaniasis. Increasing resistance and severe side effects of existing drugs have led to the need to identify new chemotherapeutic targets. Aminoacyl-tRNA synthetases (aaRSs) are ubiquitous and are required for protein synthesis. aaRSs are known drug targets for bacterial and fungal pathogens. Here, we have characterized and evaluated the essentiality of *L. donovani* lysyl-tRNA synthetase (*Ld*LysRS). Two different coding sequences for lysyl-tRNA synthetases are annotated in the *Leishmania* genome database. *Ld*LysRS-1 (LdBPK_150270.1), located on chromosome 15, is closer to apicomplexans and eukaryotes, whereas *Ld*LysRS-2 (LdBPK_300130.1), present on chromosome 30, is closer to bacteria. In the present study, we have characterized *Ld*LysRS-1. Recombinant *Ld*LysRS-1 displayed aminoacylation activity, and the protein localized to the cytosol. The *Ld*LysRS-1 heterozygous mutants had a restrictive growth phenotype and attenuated infectivity. *Ld*LysRS-1 appears to be an essential gene, as a chromosomal knockout of *Ld*LysRS-1 could be generated when the gene was provided on a rescuing plasmid. Cladosporin, a fungal secondary metabolite and a known inhibitor of LysRS, was more potent against promastigotes (50% inhibitory concentration [IC_50_], 4.19 µM) and intracellular amastigotes (IC_50_, 1.09 µM) than were isomers of cladosporin (3-epi-isocladosporin and isocladosporin). These compounds exhibited low toxicity to mammalian cells. The specificity of inhibition of parasite growth caused by these inhibitors was further assessed using *Ld*LysRS-1 heterozygous mutant strains and rescue mutant promastigotes. These inhibitors inhibited the aminoacylation activity of recombinant *Ld*LysRS. Our data provide a framework for the development of a new class of drugs against this parasite.

**IMPORTANCE** Aminoacyl-tRNA synthetases are housekeeping enzymes essential for protein translation, providing charged tRNAs for the proper construction of peptide chains. These enzymes provide raw materials for protein translation and also ensure fidelity of translation. *L. donovani* is a protozoan parasite that causes visceral leishmaniasis. It is a continuously proliferating parasite that depends heavily on efficient protein translation. Lysyl-tRNA synthetase is one of the aaRSs which charges lysine to its cognate tRNA. Two different coding sequences for lysyl-tRNA synthetases (*Ld*LysRS) are present in this parasite. *Ld*LysRS-1 is closer to apicomplexans and eukaryotes, whereas *Ld*LysRS-2 is closer to bacteria. Here, we have characterized *Ld*LysRS-1 of *L. donovani*. *Ld*LysRS-1 appears to be an essential gene, as the chromosomal null mutants did not survive. The heterozygous mutants showed slower growth kinetics and exhibited attenuated virulence. This study also provides a platform to explore *Ld*LysRS-1 as a potential drug target.

## Abstract (precis)

Aminoacyl-tRNA synthetases are housekeeping enzymes essential for protein translation, providing charged tRNAs for the proper construction of peptide chains. These enzymes provide raw materials for protein translation and also ensure fidelity of translation. *L. donovani* is a protozoan parasite that causes visceral leishmaniasis. It is a continuously proliferating parasite that depends heavily on efficient protein translation. Lysyl-tRNA synthetase is one of the aaRSs which charges lysine to its cognate tRNA. Two different coding sequences for lysyl-tRNA synthetases (*Ld*LysRS) are present in this parasite. *Ld*LysRS-1 is closer to apicomplexans and eukaryotes, whereas *Ld*LysRS-2 is closer to bacteria. Here, we have characterized *Ld*LysRS-1 of *L. donovani*. *Ld*LysRS-1 appears to be an essential gene as the chromosomal null mutants did not survive. The heterozygous mutants showed slower growth kinetics and exhibited attenuated virulence. This study also provides a platform to explore *Ld*LysRS-1 as a potential drug target.

## INTRODUCTION

Leishmaniasis is a vector-borne disease and is caused by the protozoan parasite of the genus *Leishmania*. The parasite has a dimorphic life cycle alternating between the digestive tract of the female sand fly vector as extracellular flagellated promastigotes and the phagolysosomal compartment of mammalian macrophages as an intracellular amastigote (1). Visceral leishmaniasis (VL) caused by *Leishmania donovani* is the severe form and is potentially fatal. Due to the lack of an effective vaccine against the disease, VL treatment primarily relies on chemotherapy (2). Moreover, the emergence of resistance to the currently available drugs (3) has worsened the situation. Hence, there is an urgent need to identify novel drug targets to control this disease.

Aminoacyl-tRNA synthetases (aaRSs) are essential enzymes in protein translation, ligating specific amino acids to their cognate tRNAs (4). These enzymes catalyze a two-step process in which the amino acid is activated by formation of an enzyme-bound aminoacyl-adenylate intermediate followed by the transfer of the activated amino acid to either the 2′-OH or the 3′-OH on the 3′-terminal adenosine of the tRNA (5). The aaRSs can be divided into two classes (class I and class II) based on distinct catalytic domain architectures with exclusive signature motifs for ATP binding (5). Aminoacyl-tRNA synthetases have been a focus of research against the eukaryotic parasites (6). If these enzymes are inhibited, protein translation is halted, which in turn results in the attenuation of parasite growth.

Lysyl-tRNA synthetases (LysRS) are unique as they are found as both class I and class II enzymes (7). Class II LysRS is present in all eukaryotes and most prokaryotes, while class I LysRS has been seen in few bacteria and most archaea (8, 9). The class I synthetases contain conserved HIGH and KMSKS residues in the active site. Human LyRS belongs to class II aminoacyl-tRNA synthetases as it lacks both these conserved sequences. The canonical function of LysRS (like that of other aaRSs) is to ligate l-lysine to cognate tRNAs. Besides this, these synthetases can carry out many noncanonical functions like rRNA biogenesis, angiogenesis, apoptosis, transcriptional regulation, and cell signaling in both humans and parasites (10–13).

LysRS from various organisms like *Entamoeba histolytica* have been reported to contain a chemokine that imitates the sequence, structure, and role of the human cytokine *Hs*EMAPII (*Homo sapiens* endothelial monocyte-activating polypeptide II) (14). In *Plasmodium falciparum*, LysRS have been documented to modulate a variety of cellular functions by synthesizing signaling molecules like diadenosine polyphosphates (15). In *Trypanosome brucei*, there are two copies of LysRS (*Tb*LysRS-1 and *Tb*LysRS-2). Both the copies are encoded by the nuclear genome. There is a strict functional segregation of the cytosolic and mitochondrial LysRS. The presence of a C-terminal extension in *Tb*LysRS-2 helps the enzyme to remain inactive in the cytosol, but once this enzyme is translocated to mitochondria, the C-terminal sequence is cleaved to produce a mature and active enzyme (16). Crystal structure and functional analysis of human LysRS have revealed that this enzyme can be present in dimeric and tetrameric forms, where the tetrameric form is active during translation and the dimeric form participates in the regulation of transcription (17, 18). Previous reports indicate that cladosporin, a fungal secondary metabolite, inhibits LysRS of *P. falciparum* with high potency (19). Also, LysRS from tropical worm parasites *Loa loa* (nematode) and *Schistosoma mansoni* (flatworm) showed 60-fold-better binding with cladosporin than did a human enzyme (20).

Our previous *in silico* analysis led to the identification of a total of 26 aaRSs in *Leishmania* (21). The *Leishmania* genome encodes two copies of *Ld*LysRS (TriTrypDB identifiers [IDs] LdBPK_150270.1 and LdBPK_300130.1). The gene present on chromosome 15 encodes 586-amino-acid-long *Ld*LysRS-1, and the gene present on chromosome 30 encodes 536-amino-acid-long *Ld*LysRS-2. *Ld*LysRS-1 belongs to the class II synthetases. In the present study, we for the first time report the molecular and enzymatic characterization of the LysRS-1 enzyme from *Leishmania donovani*. The physiological role of *Ld*LysRS-1 was elucidated by making gene deletion mutations using targeted gene replacement methodology. Heterozygous knockout mutants of *Ld*LysRS-1 showed reduced growth and were attenuated in their infectivity, indicating the essentiality of this protein. Cladosporin, a fungal secondary metabolite, and 3-epi-isocladosporin, an isoform of isocladosporin (22, 23), showed antileishmanial activity in both the promastigote and intracellular amastigote stages *in vitro*. Both drugs were found to be effective in inhibiting the aminoacylation activity of the recombinant *Ld*LysRS-1. In sum, the data show that *Ld*LysRS-1 is essential for the survival of *L. donovani* and can be used as a drug target.

## RESULTS

### Sequence and phylogenetic analysis.

**FIG 1:**
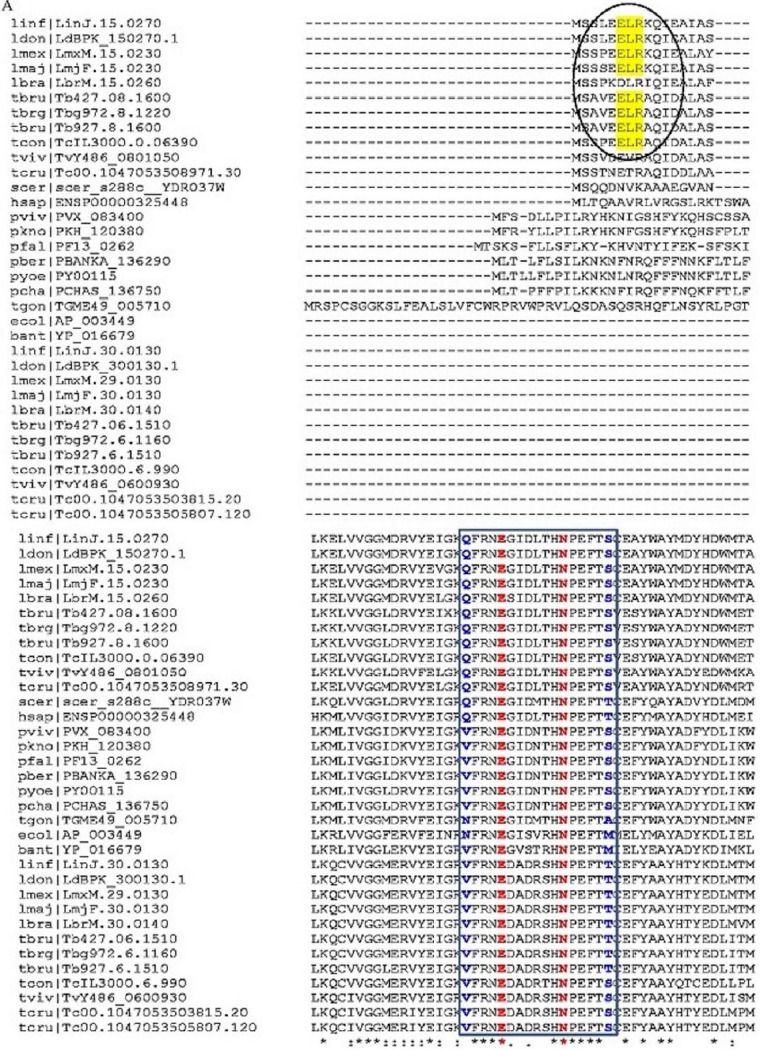

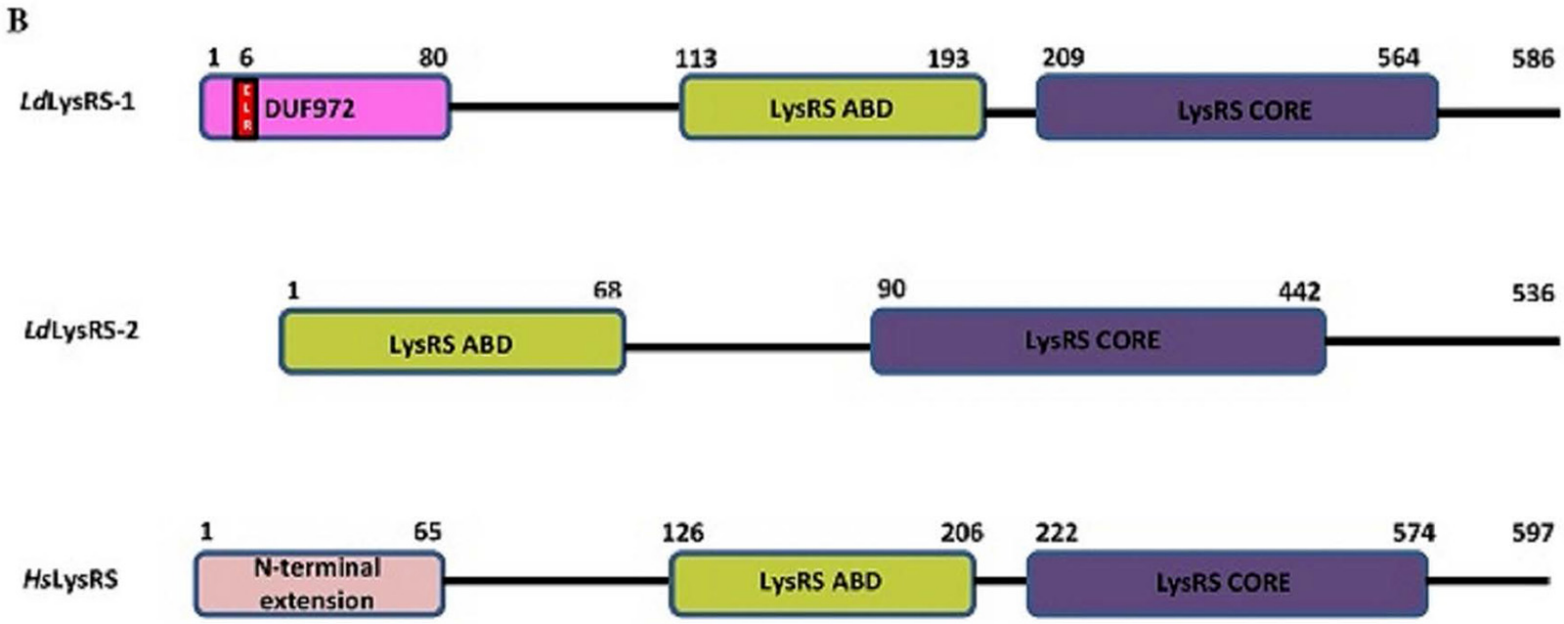
(A) Multiple sequence alignment of representative LysRS sequences from kinetoplastids, humans, yeast, plasmodia, and bacterial species generated using Clustal W (35). The ELR motif is highlighted in yellow. The key residues present in the ATP-binding site are highlighted in blue and red. For analysis, we used Linj.15.0270, LdBPK_150270.1, LmxM.15.0230, LmjF.15.0230, LbrM.15.0260, Tb427.08.1600, Tbg972.8.1220, Tb927.8.1600, TcIL3000.0.06390, TvY486_0801050, Tc00.1047053508971.30, scer_s288c_YDR037w, ENSP00000325448, PVX_083400, PKH_120380, PF13_0262, PBANKA_136290, PY00115, PCHAS_136750, TGME49_005710, AP_003449, YP_016679, Linj.30.0130, LdBPK_300130.1, LmxM.29.0130, LbrM.30.0140, Tb427.06.1510, Tbg972.6.1160, Tb927.6.1510, TcIL3000.6.990, TvY486_0600930, Tc00.1047053503815.20, and Tc00.1047053505807.120. (B) Domain architecture of *Ld*LysRS-1, *Ld*LysRS-2, and *Hs*LysRS protein. The catalytic core domain (CORE) and anticodon binding domains (ABD) are indicated. The ELR motif is present at the N terminus of *Ld*LysRS-1 and is shown in red. The N-terminal extension is present in *L. donovani Ld*LysRS-1 (domain of unknown function, DUF972) and human *Hs*LysRS protein. Its function is unknown in *Leishmania*.

In common with *Trypanosoma*, two LysRS sequences were identified in the *Leishmania* genome database (EuPath.db.org). In *Leishmania donovani*, LysRS-encoding genes are present on chromosomes 15 (LysRS-1) (TriTrypDB ID LdBPK_150270.1) and 30 (LysRS-2) (TriTrypDB ID LdBPK_300130.1). *Ld*LysRS-2 is closer to *Tb*LysRS-2 and has a C-terminal extension similar to that of *Tb*LysRS-2 (Fig. 1). Multiple sequence alignment of the kinetoplastid LysRS homologs with representative sequences from other eukaryotes (such as humans, yeast, and *Plasmodium*) and archaea (Fig. 1A) suggests the conservation of important ATP-binding residues that are essential for the functioning of the enzyme. The presence of an ELR (Glu-Leu-Arg) motif in LysRS-1 has already been reported in *Leishmania major* (21). This motif is the signature motif conserved among CXC chemokines (24). The alignment showed conservation of the ELR motif in only one of the LysRS sequences in both *Leishmania* and *Trypanosoma*. A comparison of the domain architectures of *Ld*LysRS-1, *Ld*LysRS-2, and human LysRS (*Hs*LysRS) is shown in Fig. 1B. *Ld*LysRS-1 has an N-terminal extension of 80 amino acids (DUF972) with an ELR motif. *Hs*LysRS also has a 65-amino-acid N-terminal extension. The N-terminal extension in mammals has been reported to participate in tRNA binding (25), whereas its role in *Leishmania* is not known.

**FIG 2:**
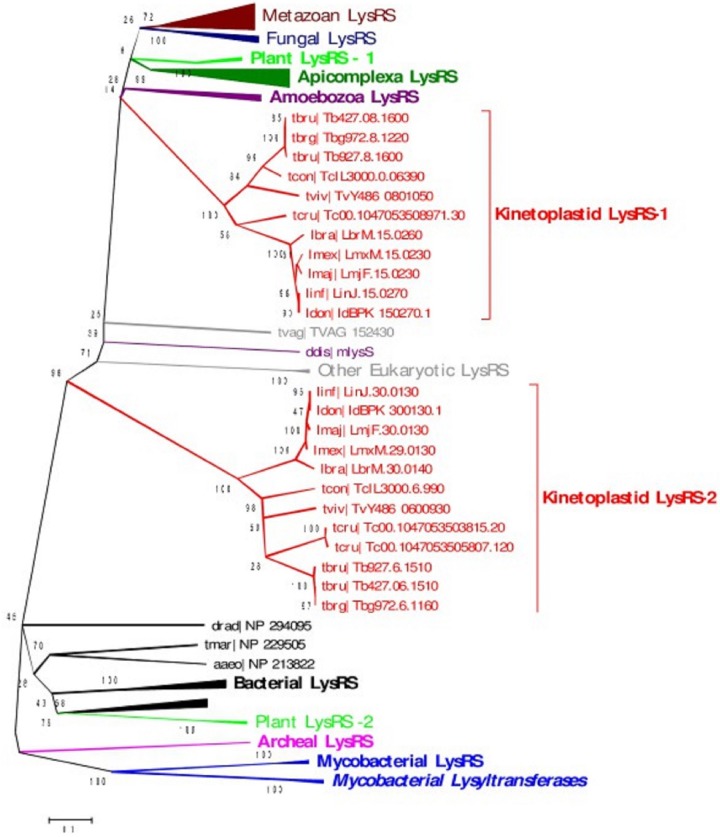
Sequence-based phylogeny of LysRS homologs from kinetoplastids, apicomplexans, metazoans, and fungal, plant, and archaeal species. The neighbor joining bootstrap tree was constructed using MEGA v5 (36). Bootstrap values of >90 are shown in the phylogenetic tree.

A phylogenetic tree was constructed using the LysRS homologs from kinetoplastids, apicomplexans, metazoans, and fungal, plant, bacterial, and archaeal species. LysRS-2 is phylogenetically closer to bacterial LysRS while LysRS-1 is closer to LysRS of apicomplexans and other eukaryotes (Fig. 2).

### Cloning, overexpression, purification, and evaluation of the oligomeric state of *Ld*LysRS-1.

**FIG 3:**
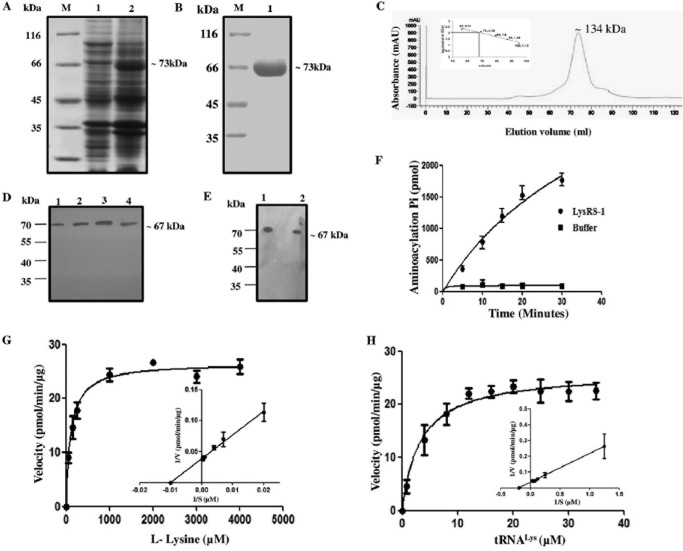
Protein induction, purification, and enzymatic characterization of recombinant *Ld*LysRS-1. (A) SDS-PAGE analysis of whole-cell lysate of uninduced and induced *E. coli* BL21(DE3) cells transformed with pET-30a–*Ld*LysRS-1. M, molecular mass marker; lane 1, uninduced bacterial cell lysate; lane 2, induced bacterial cell lysate. (B) Purification of r*Ld*LysRS-1 protein on Ni^2+^-NTA affinity resin. M, molecular mass marker; lane 1, eluted fraction with 100 mM imidazole showing purified r*Ld*LysRS-1. (C) GPC elution profile of purified *Ld*LysRS-1. Comparison with standard markers indicates that *Ld*LysRS-1 elutes at a size corresponding to the dimeric state. mAU, milli-absorbance unit. (D) Western blot analysis of the r*Ld*LysRS-1 protein and promastigote cell lysates of wild-type (WT) parasites using anti-*Ld*LysRS-1 antibody. Lane 1, 0.5 μg r*Ld*LysRS-1 protein; lane 2, 1 μg r*Ld*LysRS-1 protein; lane 3, 2 μg r*Ld*LysRS-1 protein; lane 4, *Leishmania* promastigote cell lysate (~40 μg). (E) Western blot analysis of the r*Ld*LysRS-1 protein and amastigote cell lysates of WT parasites. Lane 1, 2 μg recombinant *Ld*LysRS-1 protein; lane 2, *Leishmania* amastigote cell lysate (~40 μg). (F) Time course of tRNA^Lys^ aminoacylation by recombinant *Ld*LysRS-1. Reactions were performed with l-lysine and tRNA^Lys^ as the substrates. The data show an average from three experiments performed in duplicate ± SD. (G and H) Aminoacylation kinetics of *Ld*LysRS-1 as a function of l-lysine concentration (G) and tRNA^Lys^ concentration (H). The results represent means ± SD (*n* = 3).

The full-length *LdLysRS-1* gene was cloned into a pET-30a expression vector in order to characterize the protein. An induction of His_6_-tagged *Ld*LysRS-1 protein with an estimated molecular mass of ~73 kDa was observed (Fig. 3A). This size correlated with the amino acid composition of *Ld*LysRS-1 (~67 kDa) with a His_6_ tag (~6 kDa) at the N terminus (Fig. 3A). Recombinant *Ld*LysRS-1 (r*Ld*LysRS-1) was purified by metal affinity chromatography (Fig. 3B). In order to assess the oligomeric state of the *Ld*LysRS-1 protein, we performed gel permeation chromatography (GPC) (Fig. 3C) using a standardized column with known standards. In our GPC experiment, we observed that LysRS-1 eluted at a size corresponding to the predicted dimers (Fig. 3C), unlike the human LysRS, which displays a tetrameric form (17). The recombinant *Ld*LysRS-1 protein was analyzed by matrix-assisted laser desorption ionization–time of flight (MALDI-TOF)/TOF mass spectroscopy (data not shown). The spectrum of the protein examined by BioTool version 2.2 demonstrated intensity coverage of 44% for putative LysRS-1 (*Leishmania infantum* JPCM5). The expression of the full-length *Ld*LysRS-1 was confirmed in *Leishmania* promastigote and amastigote cell lysates by immunoblotting (Fig. 3D and E). The anti-*Ld*LysRS-1 antibody detected an ~67-kDa band in the cell extracts of both the promastigotes (Fig. 3D, lane 4) and amastigotes (Fig. 3E, lane 2).

### Enzymatic activity and kinetic parameters for *Ld*LysRS-1.

A coupled-enzyme assay was performed to assess the aminoacylation activity of r*Ld*LysRS-1. The aminoacylation reaction was carried out with r*Ld*LysRS-1 in the presence of inorganic pyrophosphatase (PPiase), and the P_i_ produced in the reaction was measured using malachite green solution. Figure 3F shows that r*Ld*LysRS-1 acylated tRNA^Lys^ in a time-dependent manner, demonstrating that the *L. donovani LysRS* gene encodes a functional enzyme. The kinetic parameters of *Ld*LysRS-1 were established utilizing l-lysine and tRNA^Lys^ as the substrates. The effect of different concentrations of l-lysine was examined while other constituents were kept constant (Fig. 3G). The *K*_*m*_ value of r*Ld*LysRS-1 for l-lysine was 111 ± 15 μM, which is closer to that documented in the case of humans (25). Since tRNA^Lys^ is another essential substrate for the aminoacylation reaction, we, therefore, performed analysis of tRNA^Lys^-dependent enzyme kinetics (Fig. 3H). The estimated *K*_*m*_ of *Ld*LysRS for tRNA^Lys^ was 3.33 ± 0.80 μM.

### Subcellular localization of *Ld*LysRS-1.

**FIG 4:**
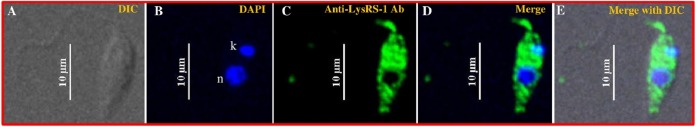
Subcellular localization of *Ld*LysRS-1 in *L. donovani*. Immunofluorescence analysis by confocal micrographs of wild-type log-phase promastigotes. (A) Phase-contrast image. DIC, differential interference contrast. (B) Promastigotes stained with DAPI. (C) Anti-*Ld*LysRS-1 antibody (Ab) detected using Alexa 488 (green)-conjugated secondary antibody. (D and E) Merged micrographs of panels B and C. “k” and “n” indicate kinetoplastid and nuclear DNA, respectively. Bar, 10 µm.

Our earlier studies using web-based prediction of signal sequences using PSORT-II indicated cytosolic localization of *Ld*LysRS-1 (21). The localization of LysRS-1 in *L. donovani* was ascertained by immunofluorescence analysis of log-phase promastigotes using an anti-*Ld*LysRS-1 antibody and 4′,6-diamidino-2-phenylindole (DAPI). Figure 4B shows the kinetoplast (k) and nuclear DNA (n) as indicated by the bright staining with DAPI. Analysis by confocal microscopy revealed that *Ld*LysRS is localized in the cytosol of the parasites (Fig. 4C). The mouse preimmune sera, nonpermeabilized cells, and secondary antibody were used as controls. No detectable signal was detected with this control (data not shown).

### Gene deletion of *Ld*LysRS-1.

**FIG 5:**
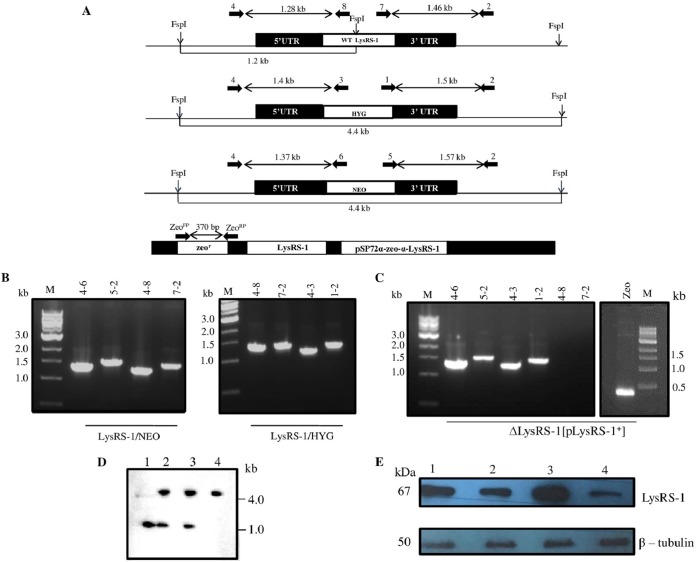
Generation of genetically modified mutants of *LdLysRS-1*. (A) Restriction map of the *LdLysRS-1* genomic locus (*LysRS-1* allele, linear replacement cassettes, containing HYG and NEO) and a genomic map of the pSP72α-zeo-α-*LysRS-1* episomal construct. The primer pairs used to assess the genotypes of mutants by PCR-based analysis along with the expected band sizes are marked. (B) PCR analysis of heterozygous (*LysRS-1/HYG* and *LysRS-1/NEO*) mutant parasites to evaluate the specific integration of the replacement cassette(s) by using *HYG*, *NEO*, and *LdLysRS-1* (WT) gene-specific primers. (C) *ΔLysRS-1*[*pLysRS-1*^*+*^] mutant parasites were used as a template for PCR analysis. The specific integration of the replacement cassette(s) was checked with *HYG*, *NEO*, and *LdLysRS-1* (WT) gene-specific primers. Zeo denotes amplification using zeocin-specific primers for detection of a pSP72α-zeo-α-*LysRS-1* episome. M indicates the molecular size markers in kilobases. Lane numbers in panels B and C indicate the primers used for each lane. (D) Southern blot analysis of genomic DNA from wild-type (WT) (lane 1), heterozygous mutant (*LysRS-1/HYG*) (lane 2), *LysRS-1/NEO* (lane 3), and *ΔLysRS-1*[*pLysRS-1*^*+*^] (lane 4) parasites. Genomic DNA was digested with FspI and separated on a 0.7% agarose gel for Southern blot analysis. Molecular sizes are indicated to the right of the blot. (E) Western blot analysis of equal protein quantities (~30 μg) from whole-cell lysates. Lane 1, WT; lane 2, add-back (*LysRS-1*/*HYG*[*pLysRS*^*+*^]); lane 3, *LysRS-1* overexpressors (WT[*pLysRS-1*^+^]); lane 4, heterozygous mutant (*LysRS-1*/*HYG*) parasites. The loading was normalized with β-tubulin (50-kDa) antibody.

In order to determine the indispensability of *LdLysRS-1* in the parasite, classical gene replacement experiments were employed, where efforts were made to replace both the wild-type (WT) alleles of *LdLysRS-1* with cassettes harboring drug resistance marker genes. As elucidated in Materials and Methods, this was done by the generation of inactivation cassettes having hygromycin phosphotransferase (*HYG*) or neomycin phosphotransferase (*NEO*) as a selection marker fused with the flanking 5′ untranslated region (UTR) and 3′ UTR of the *LdLysRS-1* gene (Fig. 5A). Linear replacement cassettes were prepared by PCR-based fusion reactions and were electroporated into the wild-type (WT) *L. donovani* promastigotes. This resulted in the generation of heterozygous parasites (*LysRS-1/HYG* or *LysRS-1/NEO*) in which either the hygromycin or neomycin drug resistance gene replaced one allele of the *LdLysRS-1* gene. Further, the PCR-based analysis was done to confirm the genotype of the heterozygous parasites (*LysRS-1/HYG* or *LysRS-1/NEO*) by utilizing primers (Table 1) external to the linear replacement cassette of the *LdLysRS-1* gene (Fig. 5A). The correct integration of *HYG* and *NEO* replacement cassettes at the *LdLysRS-1* locus was observed as indicated in Fig. 5B. Insertion of the *HYG* cassette resulted in the appearance of a 1.4-kb band (Fig. 5B, lane 4-3) and a 1.5-kb band (Fig. 5B, lane 1-2). Insertion of the *NEO* cassette at the *LdLysRS-1* locus is indicated by the presence of a 1.37-kb band (Fig. 5B, lane 4-6) and a 1.57-kb band (Fig. 5B, lane 5-2). The presence of the WT allele was confirmed by the appearance of a 1.28-kb band (Fig. 5B, lane 4-8) and a 1.46-kb band (Fig. 5B, lane 7-2). This confirmed the replacement of one allele of the *LdLysRS-1* gene in the heterozygous parasites (*LysRS-1/HYG* or *LysRS-1/NEO*). The heterozygous parasites (*LysRS-1/HYG* or *LysRS-1/NEO*) were then electroporated with a second cassette to replace the second allele of the *LdLysRS-1* gene. Several attempts to replace both the alleles of the *LdLysRS-1* gene failed, thus indicating the essentiality of *LdLysRS-1* in the *Leishmania* parasite.

**TABLE 1:** Primers used for molecular characterization of genetically manipulated *L. donovani* by PCR-based analysis

Primer no.	Primer name	Sequence
1	Primer 1	5′ TGTAGAAGTACTCGCCGATAGTGG 3′
2	Primer 2	5′ ACTCGGAACTTGGCAGAGTGTGCAC 3′
3	Primer 3	5′ CGCAGCTATTTACCCGCAGGACAT 3′
4	Primer 4	5′ TGGACGGGCTCCAGAGAGAATTCAGG 3′
5	Primer 5	5′ ATAGCGTTGGCTACCCGTGATATTGC 3′
6	Primer 6	5′ AACACGGCGGCATCAGAGCAGCCGATTG 3′
7	Primer 7	5′ ATGGTCAGGGTGTTCCCCTGCTGTAG 3′
8	Primer 8	5′ TACGGAGCTCTTCGAGGGACGACAT 3′
9	Zeo^FP^	5′ ATGGCCAAGTTGACCAGTGCCGTTCC 3′
10	Zeo^RP^	5′ TCAGTCCTGCTCCTCGGCCACGAA 3′

To further establish the essentiality of the *LdLysRS-1* gene, the construction of homozygous null mutants was attempted in the presence of a rescuing episome that has the *LdLysRS-1* gene (pSP72α-zeo-α-*LysRS-1*). The heterozygous parasites (*LysRS-1*/*HYG*) were transfected with pSP72α-zeo-α-*LysRS-1* to generate *LysRS-1/HYG*[*pLysRS-1*^*+*^] mutants. After selection of these parasites in double-antibiotic-containing M199 medium, these mutant parasites (*LysRS-1/HYG*[*pLysRS-1*^*+*^]) were transfected with the 5′ UTR-NEO-3′ UTR construct. After 3 to 4 passages, genomic DNA was isolated and investigated for the presence of the *LdLysRS-1* gene in these *ΔLysRS-1*[*pLysRS-1*^*+*^] triple-drug-resistant parasites. PCR analysis revealed the absence of the *LdLysRS-1* gene in these parasites (Fig. 5C, lanes 4-8 and 7-2), and bands corresponding to the integration of *HYG* (Fig. 5C, lanes 4-3 and 1-2) and *NEO* (Fig. 5C, lanes 4-6 and 5-2) cassettes could be detected. The presence of an episome construct (pSP72α-zeo-α-*LysRS-1*) was confirmed using zeocin-specific primers (Fig. 5C and Table 1).

The presence of *HYG/NEO* replacement cassettes in mutants was further confirmed by Southern blot analysis. Digestion of the *LdLysRS-1* gene locus in the WT cells with FspI enzyme resulted in a 1.2-kb band after probing with the 5′ UTR of the *LdLysRS-1* gene (Fig. 5D, lane 1). The heterozygous mutants had an additional band of 4.4 kb corresponding to *HYG* and *NEO* integrations (Fig. 5D, lanes 2 and 3, respectively). In the case of *ΔLysRS-1*[*pLysRS-1*^*+*^] parasites, the band corresponding to the WT gene was absent (Fig. 5D, lane 4). However, a band corresponding to *HYG* and *NEO* integration (4.4 kb) was observed (Fig. 5D, lane 4).

The protein level of *Ld*LysRS-1 was studied across different parasitic lines by Western blotting, to see the effect of disruption of a single allele of the *LysRS-1* gene. Comparative densitometric analysis revealed 2.6-fold-lower expression of the LysRS-1 protein in heterozygous mutants (*LysRS-1*/*HYG*) (Fig. 5E, lane 4) compared to that in WT parasites (Fig. 5E, lane 1). Complementation of the heterozygous parasites (*LysRS-1*/*HYG*) with an episomal copy of the *LysRS-1* gene (*LysRS-1/HYG*[*pLysRS-1*^*+*^]) restored protein expression to levels comparable to that of WT parasites (Fig. 5E, lane 2). Overexpressing mutants (WT[*pLysRS-1*^*+*^]) were also confirmed by Western blotting. An increase in LysRS-1 protein level (3-fold) was observed in *LysRS-1* overexpressors (WT[*pLysRS-1*^*+*^]) (Fig. 5E, lane 3) compared to the WT parasites (Fig. 5E, lane 1).

**FIG 6:**
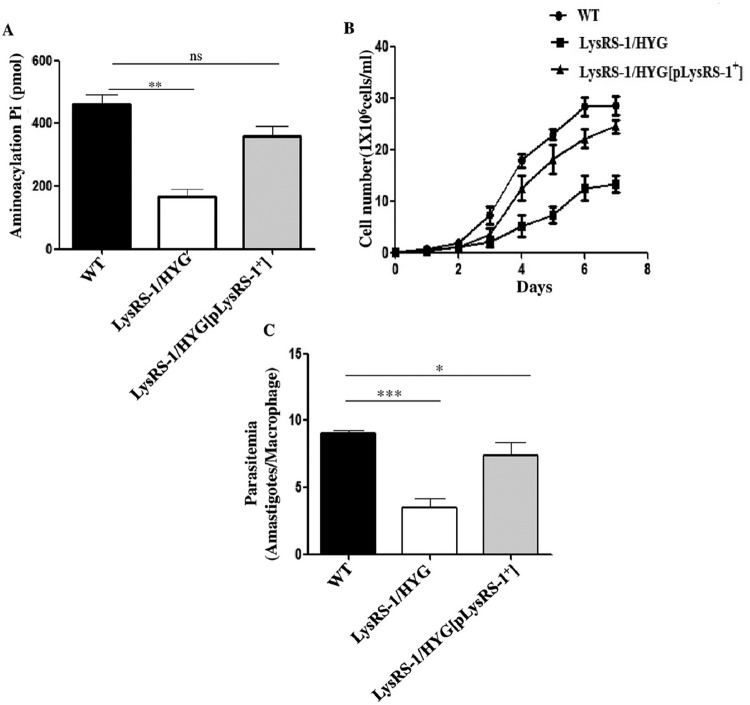
Characterization of genetically modified mutants of *LdLysRS-1*. (A) Comparison of the aminoacylation activity of LysRS-1 in cell lysates of WT, heterozygous (*LysRS-1/HYG*) mutant, and add-back (*LysRS-1/HYG*[*pLysRS-1*^+^]). (B) Growth curve of *L. donovani* WT, add-back (*LysRS-1/HYG*[*pLysRS-1*^+^]), and heterozygous mutant (*LysRS-1/HYG*) promastigotes in M199 medium. (C) Assessment of infectivity of WT, heterozygous mutant (*LysRS-1/HYG*), and add-back (*LysRS-1/HYG*[p*LysRS-1*^+^]) parasites in the THP-1 cell line. THP-1 cells were infected with stationary-phase promastigotes at an MOI of 20:1. After 24 h, cells were stained with propidium iodide and intracellular amastigotes were counted visually. The results represent means ± SD (*n* = 3). *, *P* < 0.05; **, *P* < 0.01; ***, *P* < 0.005; ns, nonsignificant data (*P* > 0.05).

The aminoacylation activity of LysRS-1 was measured in genetically modified parasites and compared to that of WT parasites (Fig. 6A). This was done to establish if the deletion of a single allele of *LysRS-1* resulted in the decrease in aminoacylation activity of *LysRS-1*. A significant reduction in the aminoacylation activity of LysRS-1 was observed in the heterozygous parasites (*LysRS-1*/*NEO*) (2.8-fold) compared to that of the WT parasites. Comparable LysRS-1 activity levels were exhibited in add-back mutants (*LysRS-1/HYG*[*pLysRS-1*^*+*^]) and the WT strain (Fig. 6A).

Analysis of the growth kinetics of heterozygous and rescue mutant parasites was undertaken to verify if the reduced expression of *LysRS-1* affects the growth of the parasites. The heterozygous parasites (*LysRS-1*/*HYG*) showed a consistent growth delay compared to their WT counterparts (Fig. 6B). Add-back mutants (*LysRS-1/HYG*[*pLysRS-1*^*+*^]) rescued the growth of these parasites similar to that of the WT control (Fig. 6B). It is possible that a gene dosage resulted in the lesser synthesis of LysRS-1 protein, thereby leading to suboptimal cell proliferation.

We also wanted to ascertain whether the heterozygous mutant parasites (*LysRS-1*/*HYG*) compromised the capability of *L. donovani* to infect host cells. THP-1 differentiated macrophages were infected with WT, *LysRS-1* heterozygous mutant (*LysRS-1*/*HYG*), or add-back (*LysRS-1/HYG*[*pLysRS-1*^*+*^]) parasites at a multiplicity of infection (MOI) of 20:1. At 24 h postinfection, parasitemia of the heterozygous mutants was reduced by ~50% compared to the WT parasites (Fig. 6C). Comparable results were obtained with *LysRS-1/NEO* parasites (data not shown). The add-back line (*LysRS-1/HYG*[*pLysRS-1*^*+*^]) showed restoration of infectivity of heterozygous mutants (*LysRS-1*/*HYG*), and the infectivity was comparable to that of the WT parasites (Fig. 6C). Our data indicate that the *LdLysRS-1* gene has a major role in the proliferation and survival of amastigotes in the macrophage.

### Leishmanicidal activity of LysRS inhibitors.

**FIG 7:**
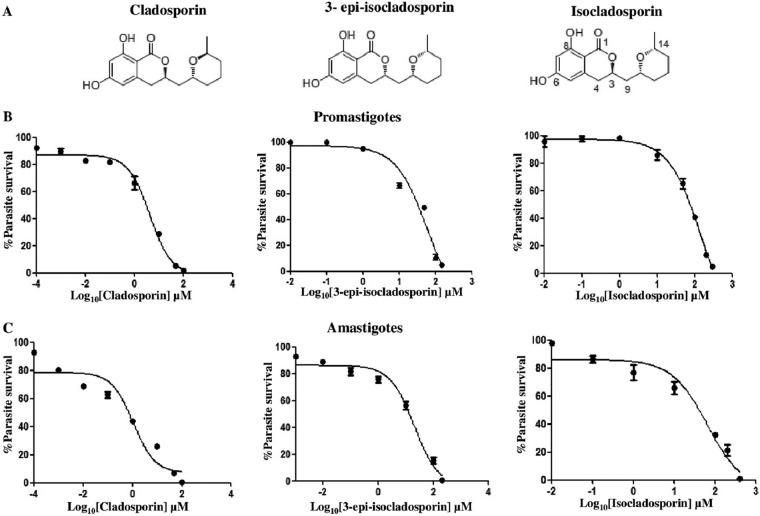
Effect of LysRS-1 inhibitors on parasite growth. (A) Chemical structure of cladosporin, isocladosporin, and 3-epi-isocladosporin. (B) Dose-response inhibition of WT promastigote growth in the presence of cladosporin, 3-epi-isocladosporin, and isocladosporin. Inhibitor concentrations are plotted on a log scale on the *x* axis. The assay was done in 96-well plates, and growth was estimated by MTT assay. Percent parasite survival was plotted against different concentrations of inhibitors. (C) The intracellular parasite load was determined using propidium iodide staining of the infected THP-1 cells, 72 h after treatment with the various concentrations of cladosporin, 3-epi-isocladosporin, and isocladosporin. The graph depicts the parasite load relative to untreated controls. The results were obtained in duplicates as representative of 2 independent experiments.

Cladosporin is a fungal secondary metabolite found in several fungi, including *Aspergillus flavus and Cladosporium cladosporioides* (19). Cladosporin has been shown to inhibit the activity of *Plasmodium* LysRS (19). Another metabolite isolated from *Cladosporium cladosporioides*, isocladosporin, exhibits antibacterial, antifungal, and plant-growth-inhibitory activity (23). Cladosporin and isocladosporin are composed of a tetrahydropyran (THP) ring (2,6-disubstituted tetrahydropyran) and a δ-valerolactone with a fused 1,3-dihydroxybenzene ring (Fig. 7A). 3-Epi-isocladosporin (Fig. 7A) is an isomer of isocladosporin (23). To test the efficacy of these compounds on *L. donovani*, WT log-phase promastigotes were cultured with increasing concentrations of these compounds. The concentrations of drugs which caused 50% inhibition of promastigote growth (IC_50_) after 72 h of addition of cladosporin, 3-epi-isocladosporin, and isocladosporin were 4.2 µM, 61.7 µM, and 156 µM, respectively (Fig. 7B). We also examined the survival of amastigotes inside macrophages in the presence of these three compounds (Fig. 7C). The IC_50_s of cladosporin, 3-epi-isocladosporin, and isocladosporin for amastigotes after 3 days of drug treatment were 1.1 µM, 20.1 µM, and 65.9 µM, respectively. At these concentrations, all three compounds did not affect the viability of the THP-1 differentiated macrophage cell line. The CC_50_s (50% cytotoxic concentrations) of cladosporin, 3-epi-isocladosporin, and isocladosporin for macrophages were 113 µM, 200 µM, and 200 µM, respectively.

**FIG 8:**
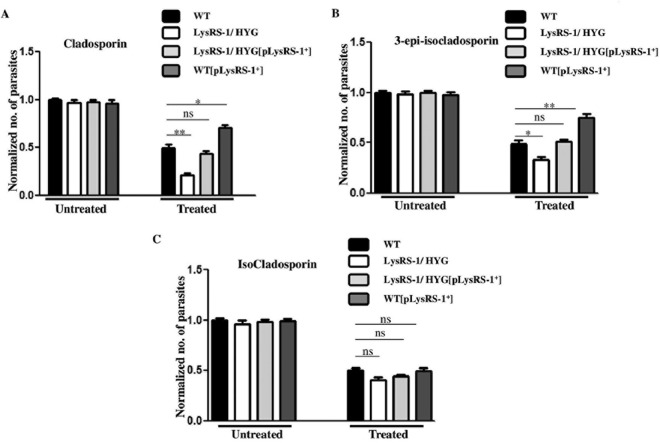
Inhibition profile of cladosporin, 3-epi-isocladosporin, and isocladosporin for the promastigote growth of WT and genetically manipulated parasites. WT, overexpressor (WT[*pLysRS-1*^*+*^]), heterozygous mutant (*LysRS-1*/*HYG*), and add-back (*LysRS-1/HYG*[*pLysRS-1*^*+*^]) parasites were treated with cladosporin (A), 3-epi-isocladosporin (B), and isocladosporin (C) with concentrations above their IC_50_s: 5 μM, 65 μM, and 160 μM, respectively. The cell growth was determined after 72 h of drug treatment. In the absence of drug treatment (Untreated), the growth of each parasitic line was normalized to 1.0. After treatment with each drug (Treated), growth was calculated relative to the corresponding untreated control. The bar graph represents the mean ± SD (*n* = 3). *, *P* < 0.05; **, *P* < 0.01; ***, *P* < 0.005; ns, nonsignificant data (*P* > 0.05).

We evaluated the effect of cladosporin, 3-epi-isocladosporin, and isocladosporin on the growth of genetically manipulated parasites in order to ascertain whether the antileishmanial effect of these inhibitors is mediated through the inhibition of *Ld*LysRS-1. WT, heterozygous mutant (*LysRS-1*/*HYG*), add-back (*LysRS-1/HYG*[*pLysRS-1*^*+*^]), and overexpressor (WT[*pLysRS-1*^*+*^]) parasites were treated with either 5 µM cladosporin, 65 µM 3-epi-isocladosporin, or 160 µM isocladosporin. In the untreated parasites, the growth of each parasitic line was normalized to a value of 1.0. The rate of growth of each parasitic line was calculated relative to the untreated control after 72 h of treatment. Parasites overexpressing *Ld*LysRS-1 (WT[*pLysRS-1*^*+*^]) were found to be more resistant to growth inhibition by cladosporin (Fig. 8A) and 3-epi-isocladosporin (Fig. 8B), while no change in growth inhibition was seen in the case of isocladosporin (Fig. 8C). In contrast, heterozygous mutants (*LysRS-1*/*HYG*) were found to be more susceptible to inhibition by cladosporin than by 3-epi-isocladosporin, while WT parasites were about equally susceptible to the two drugs but less susceptible than the heterozygous mutants (Fig. 8A and B, respectively). The growth of heterozygous mutants (*LysRS-1*/*HYG*) was reduced by ~58% relative to the WT parasites after treatment with cladosporin, while 3-epi-isocladosporin showed a reduction of ~32% (Fig. 8A and B, respectively). Add-back mutants (*LysRS-1/HYG*[*pLysRS-1*^*+*^]) showed decreased sensitivity of parasites to cladosporin and 3-epi-isocladosporin (Fig. 8A and B, respectively). However, no change was observed when WT, heterozygous mutant (*LysRS-1*/*HYG*), add-back (*LysRS-1/HYG*[*pLysRS-1*^*+*^]), and overexpressor (WT[*pLysRS-1*^*+*^]) parasites were treated with isocladosporin (Fig. 8C). The increased susceptibility of the heterozygous mutants (*LysRS-1*/*HYG*) to cladosporin and 3-epi-isocladosporin may be explained by the reduced levels of LysRS-1 expression in the heterozygous parasites.

### Drug binding and inhibition of recombinant *Ld*LysRS-1.

**FIG 9:**
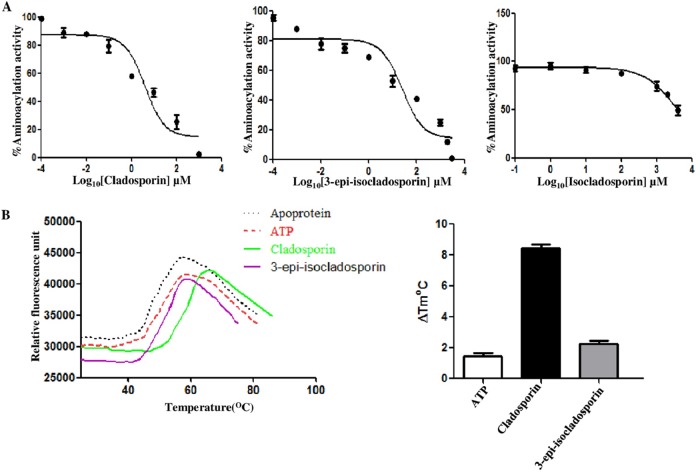
(A) Dose-response inhibition of the aminoacylation activity of *Ld*LysRS-1 in the presence of inhibitors (cladosporin, 3-epi-isocladosporin, and isocladosporin). The reaction mixture containing *Ld*LysRS-1 was incubated with different concentrations of inhibitor (0.1 nM to 1 mM) for 30 min at 37°C followed by quantitation with malachite green. (B) Thermal melting profile of *Ld*LysRS-1 protein without inhibitor (Apoprotein) and in the presence of inhibitor and ATP is shown. Results from thermal shift analysis of r*Ld*LysRS-1 in the presence of ATP, cladosporin, and 3-epi-isocladosporin are expressed in melting temperature (*T*_*m*_) variation (ΔTm°C), determined as *T*_*m*_ (protein with a ligand) − *T*_*m*_ (protein without ligand).

We also checked the effect of these compounds on the aminoacylation activity of recombinant *Ld*LysRS-1 (Fig. 9A). Cladosporin inhibited the enzymatic activity of r*Ld*LysRS-1 with an IC_50_ of ~4.07 µM, while 3-epi-isocladosporin inhibited r*Ld*LysRS-1 with an IC_50_ of ~25.5 µM. A concentration of isocladosporin as high as 1 mM failed to inhibit the enzymatic activity of *Ld*LysRS-1 (Fig. 9A). The binding of cladosporin or 3-epi-isocladosporin and *Ld*LysRS-1 was further established by checking the relative binding affinities of cladosporin, 3-epi-isocladosporin, or ATP for *Ld*LysRS-1 by performing thermal shift assays. The thermal melting profile of *Ld*LysRS-1 was only slightly altered by ATP with a shift of ~1.5°C (Fig. 9B). In contrast, addition of cladosporin and 3-epi-isocladosporin shifted the melting curve by ~8°C and ~3°C, respectively (Fig. 9B). These data indicate higher affinity and greater thermal stability of the *Ld*LysRS-1–cladosporin complex than the *Ld*LysRS-1–3-epi-isocladosporin complex.

## DISCUSSION

Aminoacyl-tRNA synthetases (aaRSs) are essential enzymes of the protein translation machinery that ensure fidelity in the translation of mRNA. These enzymes are the validated targets for the development of new antiparasitic agents with novel mechanisms of action (6). Among all aaRSs, lysyl-tRNA synthetase (LysRS) is unusual because it belongs to either class I or class II enzymes (26). In most organisms, LysRS is present as the class II form. However, in many archaea, in several alphaproteobacteria, and in spirochetes, a very different type of LysRS which is homologous to class I aaRSs is present (27).

While humans possess a single copy of LysRS (28), *Leishmania* and trypanosomes encode two copies of LysRS (21, 29). One of the *Ld*LysRS (LdBPK_150270.1) (*Ld*LysRS-1) has an N-terminal extension of 80 amino acids (DUF972). The N-terminal extension in mammals has been reported to participate in tRNA binding (25), whereas its role in *Leishmania* is not known. The N-terminal extension of *Ld*LysRS-1 also contains an ELR motif that is known to have chemokine activity in humans. *Ld*LysRS-2 has a C-terminal extension similar to that reported in *Tb*LysRS-2. This C-terminal extension in *Tb*LysRS-2 enables the enzyme to remain inactive in the cytosol, but once the enzyme is translocated to the mitochondria, the C-terminal sequence is cleaved to produce a mature and active enzyme (16). However, experimental verification of the role of N- and C-terminal extensions and the ELR motif in *L. donovani* is required to address this. We checked the triggering of cytokine secretion by a murine macrophage cell line using recombinant *Ld*LysRS-1. The culture supernatants were analyzed for the presence of proinflammatory cytokines. Time kinetic analysis by enzyme-linked immunosorbent assay (ELISA) revealed no trigger of cytokine release from macrophages (data not shown). These data indicate that *Ld*LyRS-1 is probably not a chemokine.

Earlier reports using mass spectrometry analysis of purified mitochondria and whole cells show both cytosolic and mitochondrial localization of LysRS-1 (TriTrypDB ID Tb927.8.1600) in *T. brucei* (29). In *T. brucei*, *Tb*LysRS-1 (Tb927.8.1600) was reported to be present both in the cytosol and in the mitochondria while *Tb*LysRS-2 (Tb927.6.1510) was present as a mitochondrial protein (29). In *L. donovani*, both the genes encoding LysRS (LdBPK_150270.1 and LdBPK_300130.1) are predicted to be cytosolic (21). Our immunofluorescence analysis of log-phase promastigotes confirms the cytosolic localization of LysRS-1.

In the present study, we for the first time report the molecular characterization of *Ld*LysRS-1. This study provides genetic validation of *Ld*LysRS-1 as an essential enzyme in *Leishmania*. The open reading frame (ORF) of *Ld*LysRS-1 encodes a 586-amino-acid-long polypeptide. Kinetic analysis of the recombinant *Ld*LysRS-1 showed that it exhibited catalytic efficiency similar to that reported for other mammalian LysRS (25). Our results indicate that the *LdLysRS-1* gene encodes an aaRS that is present in the cytosol. Gene deletion studies stated that LysRS-1 is essential for *L. donovani* viability and may be explored as a possible antileishmanial drug target. Earlier reports show that the knockdown of expression of the gene encoding *Tb*LysRS-1 in *T. brucei* resulted in parasite growth arrest, indicating the essentiality of this gene for parasite growth (29).

Cladosporin is a fungal secondary metabolite, and its efficacy as a lysyl-tRNA synthetase inhibitor has been reported in the case of *P. falciparum* (19). Isocladosporin, isolated from the fungus *Cladosporium cladosporioides*, is also composed of a THP ring similar to cladosporin. 3-Epi-isocladosporin is an isomer of isocladosporin (23). Cladosporin mimics the adenosine part of ATP and hence interacts with the catalytic site of the LysRS (30). The basis of cladosporin selectivity has been reported earlier (19). The majority of the amino acid residues in the ATP-binding pocket are highly conserved across different species. However, a clear variation has been reported at 2 amino acid positions corresponding to *Saccharomyces cerevisiae* residue Gln324 and Thr340 (19) (Fig. 1A). In *Ld*LysRS-1 of *Leishmania* spp., these positions are occupied by Gln308 and Ser324 residues (Fig. 1A). However, in the case of *Ld*LysRS-2, these positions are occupied by Val189 and Thr205, respectively. A clear correlation has been predicted between cladosporin activity and the identity of these amino acids at these two key positions in the ATP-binding pocket (19). Reduced cladosporin potency is predicted whenever a bulkier residue, e.g., in replacement of serine with threonine, is present at position 340, as in the case of *Saccharomyces cerevisiae*. However, *P. falciparum*, which has Val328 and Ser344, has much higher potency toward cladosporin than *L. donovani Ld*LysRS-2, which has Val189 and Thr205 (*P. falciparum*, IC_50_ of 0.04 to 0.08 µM [19]; *L. donovani*, IC_50_ of 2.56 µM [19]).

We analyzed the effects of these three compounds on parasite survival and aminoacylation activity of *Ld*LysRS-1. Cladosporin, 3-epi-isocladosporin, and isocladosporin were found to inhibit parasite growth. Cladosporin was the most efficient with the lowest IC_50_s (4.2 µM in promastigotes and 1.1 µM in amastigotes) in comparison to the other two analogues. Our data show that *Ld*LysRS-1 has glutamine at position 308 and serine at position 324, and in humans, these positions are occupied by Gln321 and Thr337, respectively (Fig. 1A). Since a bulkier amino acid (Thr) is replaced in the case of humans, this possibly results in reduced cladosporin potency (19). Our data show that cladosporin has relatively higher IC_50_s in THP-1-derived macrophages (CC_50_, 113 µM) than in amastigotes (1.1 µM).

The aminoacylation activity of *Ld*LysRS-1 was inhibited by cladosporin (IC_50_, ~4.0 µM) and 3-epi-isocladosporin (IC_50_, ~25.5 µM). A concentration of isocladosporin as high as 1 mM failed to inhibit the enzymatic activity of *Ld*LysRS-1. Cladosporin possessed a 50% inhibitory concentration of 4.0 µM against *Ld*LysRS-1 (Fig. 9A), which is comparable to its activity in cellular screens (IC_50_s, 4.2 µM in promastigotes and 1.1 µM in amastigotes). These data indicate that lysyl-tRNA synthetase is the primary target within the cell. The specificity of inhibition of lysyl-tRNA synthetase by cladosporin is also supported by using *Ld*LysRS-1 heterozygous mutant strains and rescue mutant promastigotes. In conclusion, we have characterized *Leishmania* LysRS-1 and show that it is essential for parasite growth or infectivity *in vitro*. Further studies are ongoing in the laboratory to check the efficacy of these inhibitors in the *in vivo* mouse model. The inhibitors studied here may provide a framework for the development of a new class of drugs against *Leishmania* parasites.

## MATERIALS AND METHODS

### Chemicals.

All DNA-modifying enzymes and restriction enzymes were obtained from New England Biolabs. Hygromycin, zeocin, and paromomycin were attained from Sigma. pET-30a plasmid was acquired from Novagen. Protein markers and DNA ladders were obtained from New England Biolabs. *Escherichia coli* DH10β and BL21(DE3) were utilized as hosts for plasmid cloning and protein expression, respectively. Ni^2+^-nitrilotriacetic acid (NTA) agarose was purchased from Qiagen. Cladosporin, 3-epi-isocladosporin, and isocladosporin were synthesized by Debendra K. Mohapatra, CSIR-Indian Institute of Chemical Technology, Hyderabad, India (22, 23). Sypro orange dye was obtained from Sigma. The rabbit antitubulin antibody was acquired from Neomarker (Fremont, CA). Other materials utilized as part of this study were of analytical grade and were commercially available.

### Strains and culture conditions.

Promastigote cultures of the *L. donovani Bob* strain (MHOM/SD/62/1SCL2D) were kindly provided by Stephen Beverley (Washington University, St. Louis, MO). Promastigotes were maintained by weekly passages in M199 medium (Sigma) supplemented with 100 U ml^−1^ penicillin (Sigma), 100 μg ml^−1^ streptomycin (Sigma), and 5% heat-inactivated fetal bovine serum (FBS; Gibco) at 22°C. Genetically modified *LysRS* heterozygotes (*LysRS-1/HYG* and *LysRS-1/NEO*) were grown in either 200 μg ml^−1^ hygromycin or 300 μg ml^−1^ paromomycin, respectively. Parasites (WT[*pLysRS-1*^*+*^]) overexpressing LysRS-1 were cultured in 800 μg ml^−1^ zeocin. The episomally LysRS-1-complemented *LysRS-1/HYG*[*pLysRS-1*^*+*^] heterozygous promastigotes were grown in 800 μg ml^−1^ zeocin and 200 μg ml^−1^ hygromycin. *ΔLysRS-1*[*pLysRS-1*^*+*^] parasites were cultured in 800 μg ml^−1^ zeocin, 200 μg ml^−1^ hygromycin, and 300 μg ml^−1^ paromomycin. Phenotypic characterization of mutant parasites was done in drug-free medium.

The axenic amastigotes were obtained by the standard protocol as described earlier (31). THP-1, an acute monocytic leukemia-derived human cell line obtained from ATCC, was grown in RPMI 1640 medium (Sigma) supplemented with 10% FBS and antibiotics (100 units/ml penicillin and 100 μg/ml streptomycin) at 37°C with 5% CO_2_.

### Sequence and phylogenetic analysis.

LysRS sequences retrieved from TriTrypDB (32), Swiss-Prot/UniProtKB (33), and PlasmoDB (34) were used for multiple sequence alignment. Multiple sequence alignment of these sequences was done using ClustalW (35) using default parameters and utilized as seed alignment for phylogenetic tree generation utilizing the Jones-Taylor-Thornton (JTT) model. MEGA version 5.0 (36) was utilized for both analysis and visualization of the phylogenetic tree.

### Expression and purification of the recombinant *Ld*LyRS-1 protein.

In order to express the *LdLysRS-1* gene (TriTrypDB ID LdBPK_150270.1), the coding region was PCR amplified from *L. donovani* genomic DNA using a sense primer with an adjacent BamHI site (5′ AAAGGATCCATGTCGTCCCTCGAAGAGCTCCGTA 3′) and an antisense primer with an adjoining HindIII site (5′ AAAAAGCTTCTACAGCAGGGGAACACCCTGACCAT 3′). The digested 1,761-bp PCR product covering the *Ld*LysRS open reading frame (ORF) was cloned in frame into BamHI and HindIII restriction sites of pET-30a vector (Novagen). The resulting construct (*Ld*LysRS-1–pET-30a) with a His_6_ tag at the N-terminal end was transformed into the *E. coli* BL21(DE3) strain (Novagen). The protein expression of recombinant *Ld*LysRS (r*Ld*LysRS) was induced at an optical density at 600 nm (OD_600_) of 0.6 with 0.3 mM IPTG (isopropyl-β-d-thiogalactopyranoside) at 16°C for 16 h. The protein was purified by affinity chromatography using Ni^2+^-nitrilotriacetic acid agarose resin (Qiagen) by eluting with increasing concentrations of imidazole. The protein was further purified by gel permeation chromatography on a Superdex 200 16/60 GL column (GE Healthcare). Eluted fractions were checked by SDS-PAGE, and fractions were pooled and concentrated.

### Aminoacylation assays.

The *L. donovani* tRNA^Lys^ was synthesized by *in vitro* transcription from a PCR product template, having a T7 RNA polymerase promoter followed by a gene encoding the *L. donovani* tRNA^Lys^ sequence (TriTrypDB ID LinJ.10.tRNA1) and the terminal CCA sequence. The *in vitro* transcription reaction was carried out with the MEGAscript T7 polymerase kit (Ambion; Life Technologies) at 37°C for 16 h according to the manufacturer’s guidelines. Transcripts were extracted using acid phenol-chloroform (5:1) solution, pH 4.5 (Ambion; Life Technologies), and were precipitated with isopropanol (Sigma). The tRNA was folded prior to the aminoacylation reactions by heating at 70°C for 10 min, followed by the addition of 10 mM MgCl_2_ and slow cooling at room temperature (RT). The aminoacylation reaction was done in 30 mM HEPES (pH 7.5), 150 mM NaCl, 30 mM KCl, 50 mM MgCl_2_, 1 mM dithiothreitol (DTT), 200 μM ATP, 10 mM l-lysine, 8 μM tRNA^Lys^, 2 units/ml inorganic pyrophosphatase (PPiase) (Sigma), and 0.2 μM r*Ld*LysRS-1 protein at 37°C (37). The aminoacylation reaction was stopped at different time points by the addition of 10 mM EDTA and developed by addition of malachite green (Echelon Bioscience). Absorbance was measured at 620 nm with a SpectraMax M2 reader (Molecular Devices). The *K*_*m*_ and *V*_max_ for l-lysine and tRNA^Lys^ were determined by varying the concentration of l-lysine or tRNA^Lys^ in the reaction mixture while the other components were maintained in excess. For r*Ld*LyRS-1 inhibition, a reaction mixture containing r*Ld*LysRS-1 (0.2 μM) was incubated with different concentrations of cladosporin, 3-epi-isocladosporin, and isocladosporin (0.1 nM to 1 mM) for 30 min at 37°C. Reactions were stopped and quantitated as described above. The 50% inhibitory concentration (IC_50_) was determined. Using GraphPad Prism, the dose-response data were fitted to the log (inhibitor)-versus-response equation.

### Generation of molecular constructs for the substitution of *LdLysRS-1* alleles.

A targeted gene replacement strategy based on PCR fusion was employed (38) for the inactivation of the *LdLysRS-1* gene. Briefly, flanking regions of *LdLysRS-1* were PCR amplified from genomic DNA of *L. donovani* and were linked to the hygromycin phosphotransferase gene (*HYG*) or the neomycin phosphotransferase gene (*NEO*). The 5′ UTR (783 bp) and 3′ UTR (925 bp) of the *LdLysRS-1* gene were PCR amplified using primers A and B_Hyg_ or A and B_Neo_ and primers E_Hyg_ and F or E_Neo_ and F (Table 2), respectively. *NEO* and *HYG* genes were amplified from pX63-NEO and pX63-HYG templates using primers C_Neo_ and D_Neo_ and primers C_Hyg_ and D_Hyg_ (Table 2), respectively. The 5′ UTR of the *L. donovani LysRS-1* gene was then fused to either of the antibiotic resistance marker genes (*HYG*/*NEO*) by PCR utilizing primers A and D_Hyg_ or primers A and D_Neo_. 5′ UTR-marker gene-3′ UTR constructs were obtained using primers A and F by utilizing 5′ UTR-marker gene and 3′ UTR as the templates. An episomal copy of the *LdLysRS-1* gene was generated by amplification of the *LdLysRS-1* coding sequence with a sense primer possessing the XbaI site (primer 7) and antisense primer with the HindIII site (primer 8) (Table 1). After amplification of *LdLysRS-1*, the gene was cloned into the pSP72α-zeo-α vector to get the pSP72α-zeo-α-*LysRS-1* construct. All the synthesized fragments and constructs were sequenced before transfection.

**TABLE 2:** Primers used for generation of the Hyg- and Neo-specific replacement cassette fragments

Primer no.	Primer name	Sequence
1	A	5′ AACGAACCAAAGTGCCTTCGGCGAC 3′
2	B_Hyg_	5′ GGTGAGTTCAGGCTTTTTCATCCTTTTACTGTTTTGTGGTGCG 3′
3	C_Hyg_	5′ CGCACCACAAAACAGTAAAAGGATGAAAAAGCCTGAACTCACC 3′
4	D_Hyg_	5′ CGACGAAGAGAATCACAGTCATCTATTCCTTTGCCCTCGGACGAG 3′
5	E_Hyg_	5′ CTCGTCCGAGGGCAAAGGAATAGATGACTGTGATTCTCTTCGTCG 3′
6	B_Neo_	5′ CAATCCATCTTGTTCAATCATCCTTTTACTGTTTTGTGGTGCG 3′
7	C_Neo_	5′ CGCACCACAAAACAGTAAAAGGATGATTGAACAAGATGGATTG 3′
8	D_Neo_	5′ CGACGAAGAGAATCACAGTCATTCAGAAGAACTCGTCAAGAAG 3′
9	E_Neo_	5′ CTTCTTGACGAGTTCTTCTGAATGACTGTGATTCTCTTCGTCG 3′
10	F	5′ TAGAGAGCAGTTGTTCTGCTGCAG 3′

### Creation of genetically modified parasites.

After the generation of linear replacement fragments, ~2 μg of the fragment (5′ UTR-Hyg 3′ UTR or 5′ UTR-Neo 3′ UTR) was separately transfected into wild-type *L. donovani* promastigotes (38). Drug selection was carried out depending on the marker gene. In order to check for the correct integration of inactivation cassettes, the parasites resistant to antibiotic selection were further subjected to PCR-based analysis using primers shown in Table 1. To knock out the other allele of the *LysRS-1* gene, the second round of transfection was initiated. In order to check the genotype of mutants, Southern analysis was done utilizing a standard protocol (39).

The add-back line (*LysRS-1/HYG*[*pLysRS-1*^*+*^]) was generated by complementing heterozygous *LysRS-1/HYG* parasites with an episomal construct (pSP72α-zeo-α-*LysRS-1*). In order to create *LdLysRS-1*-overexpressing parasites (WT[*pLysRS*^*+*^]), the wild-type promastigotes were transfected with the episomal construct pSP72α-zeo-α-*LysRS-1*. The correct integration was confirmed by PCR (data not shown) and Western blot analysis.

### Growth and infectivity assays.

Growth rate experiments were done by seeding stationary-phase parasites at a density of 1 × 10^6^ cells/ml in drug-free M199 medium with 5% FBS in 25-cm^2^ flasks at 22°C. The growth rate of cultures was monitored microscopically at 24-h intervals for 7 days with a Neubauer hemocytometer. The experiments were repeated at least three times. For the infectivity assay, the THP-1 cell line was plated at a cell density of 5 × 10^5^ cells/well in a 6-well flat-bottom plate. THP-1 cells were treated with 0.1 µM phorbol myristate acetate (PMA; Sigma) at 37°C for 48 h to achieve differentiation into adherent, nondividing macrophages. After activation, adherent cells were infected with stationary-phase promastigotes, at an MOI of 20:1 for 6 h. Extra nonadherent promastigotes were then removed by incubating the cells for 30 s in phosphate-buffered saline (PBS). These were then maintained in RPMI 1640 medium containing 10% FBS at 37°C with 5% CO_2_. Propidium iodide staining was done to visualize the intracellular parasite load.

### Drug inhibition assays.

The 3-(4,5-dimethyl-2-thiazolyl)-2,5-diphenyl-2H tetrazolium bromide (MTT) assay was performed with *L. donovani* promastigotes in order to determine susceptibility profile of parasites against cladosporin, 3-epi-isocladosporin, and isocladosporin. Log-phase promastigote parasites (5 × 10^4^ cells/well) were seeded in a 96-well flat-bottom plate (Nunc) and incubated with different drug concentrations in M199 medium with 5% FBS at 22°C. After 72 h of incubation, 10 μl of MTT (5 mg ml^−1^) was added to each well, and the plates were further incubated at 37°C for 4 h. The reaction was stopped by the addition of 50 μl of 50% isopropanol and 20% SDS followed by gentle shaking at 37°C for 30 min. The absorbance was measured at 570 nm in a microplate reader (SpectraMax M2 from Molecular Devices). The percentage of parasite growth relative to the untreated cells at different drug concentrations was determined, and the 50% inhibitory concentration for each drug was calculated.

The sensitivities of intracellular amastigotes to cladosporin, 3-epi-isocladosporin, and isocladosporin were determined by visualization of the intracellular parasite load using propidium iodide staining of the infected THP-1 differentiated macrophages, 72 h after treatment with different concentrations of the drug.

### Thermal shift assay.

The thermal shift assay (40) was performed with r*Ld*LysRS-1. *Ld*LysRS-1 (15 µg) diluted in 30 µl buffer containing 50 mM Tris (pH 7.5), 300 mM NaCl, 5 mM MgCl_2_, 1 mM l-lysine, and 2× Sypro orange dye along with different ligands (5 mM ATP [Sigma] and 5 mM drugs) was incubated at room temperature for 10 min. The samples were then heated from 25 to 99°C at a rate of 1°C min^−1^. Fluorescence signals were monitored by the CFX96 real-time system (Bio-Rad). The assays were repeated three times independently.

### Antibody generation and Western blot analysis.

Polyclonal antibodies against highly purified recombinant *Ld*LysRS-1 were raised commercially (Merck) in rabbits. The late-log-phase promastigotes and axenic amastigotes were harvested, and the resultant cell pellets were resuspended in lysis buffer (10 mM Tris-Cl, pH 8.0, 5 mM DTT, 10 mM NaCl, 1.5 mM MgCl_2_, 0.1 mM EDTA, 0.3 mM phenylmethylsulfonyl fluoride [PMSF], and 0.5% Triton X-100). The cells were lysed by freeze-thaw cycles followed by sonication on ice. Lysates were centrifuged at 13,000 rpm, and supernatants were fractionated on a 10% SDS-PAGE gel. Proteins were then transferred onto a nitrocellulose membrane (Bio-Rad). After blocking with 5% bovine serum albumin, the membrane was probed with primary antibodies (1:3,000 dilution) and secondary horseradish peroxidase (HRP)-conjugated antibodies (1:5,000 dilution). The blot was developed using the enhanced chemiluminescence (ECL; Amersham Biosciences) kit according to the manufacturer’s protocol.

### Immunofluorescence microscopy.

For the intracellular localization of *Ld*LysRS-1 promastigotes, the cells were washed with 1× PBS and immobilized on poly-l-lysine-coated coverslips. The cells were then fixed with 4% paraformaldehyde and permeabilized in 0.5% Triton X-100, followed by incubation with the anti-*Ld*LysRS-1 antibody (1:500) for 1 h at room temperature. Cells were washed and incubated for 45 min at room temperature (RT) with Alexa 488-conjugated goat anti-rabbit IgG antibody (Thermo Fisher Scientific). The nuclear and the kinetoplastid DNA were then stained with 1 µg/ml of DAPI (Sigma) for 15 min. The fluorescence of the stained parasites was visualized by a confocal laser scanning microscope (Olympus FluoView FV1000 with PLAPON 60× O objective lenses; numerical aperture [NA], 1.42).

### Statistical analysis.

Results for aminoacylation activity in cell lysate and in the infectivity assay were shown as column data in GraphPad Prism and were analyzed using Student’s *t* test. Data are represented as means ± standard deviations (SD). A *P* value of <0.05 was accepted as an indication of statistical significance.
